# Military culture and collaborative decision-making in mental healthcare: cultural, communication and policy considerations

**DOI:** 10.1192/bjo.2023.516

**Published:** 2023-08-14

**Authors:** Emily B. H. Treichler, Samantha J. Reznik, David Oakes, Vanessa Girard, Yaara Zisman-Ilani

**Affiliations:** VA San Diego Mental Illness Research, Education and Clinical Center, San Diego, California, USA; and Department of Psychiatry, University of California, San Diego, California, USA; VA San Diego Psychosocial Rehabilitation and Recovery Center, San Diego, California, USA; and Texas Institute for Excellence in Mental Health, University of Texas at Austin, Austin, Texas, USA; VA San Diego Mental Illness Research, Education and Clinical Center, San Diego, California, USA; VA San Diego Psychosocial Rehabilitation and Recovery Center, VA San Diego, San Diego, California, USA; Department of Social and Behavioral Sciences, College of Public Health, Temple University, Philadelphia, Pennsylvania, USA; and Department of Clinical, Educational and Health Psychology, Division of Psychology and Language Sciences, University College London, London, UK

**Keywords:** Military psychiatry, rehabilitation, shared decision-making, collaborative decision-making, health communication

## Abstract

Military culture relies on hierarchy and obedience, which contradict the implementation and use of collaborative care models. In this commentary, a team of lived experience, clinical and research experts discuss, for the first time, cultural, communication and policy considerations for implementing collaborative care models in military mental healthcare settings.

Military veterans and current military service members are at a heightened risk for mental health disorders, including suicide.^1,2^ Yet, most do not receive their needed mental healthcare.^1^ Collaborative decision-making (CDM) is a value-centred approach that engages veterans and services members in mental healthcare.^3,4^ It is associated with greater treatment-seeking, engagement and treatment satisfaction and improved treatment outcomes, including symptom severity, personal recovery and quality of life.^4^ CDM redistributes power between clinicians and patients to create an equal decision-making process that prioritises personal values, self-determination and empowerment in recovery-oriented care. For example, patients may advocate to return to a previously beneficial mental health programme when symptoms recur or to realign treatment based on personal goals outside of standard symptom remission. To actualise these ideals fully, CDM addresses not only patient–provider decisions but also patient–system decisions; whether and how a treatment is considered ‘evidence-based’; and admissions and discharge policies.^4–6^ The CDM model builds on the shared decision-making approach^7^ and seeks to overcome common implementation barriers through ‘designed for dissemination’ strategies that increase generalisability and feasibility across settings and decisional contexts.^8^

Military veterans from the USA may find the CDM model particularly important. Although patriotism in the USA is high compared with other countries^9^ and attitudes towards active duty service members and veterans tend to be positive across most groups,^10^ dynamics within and without the military have an impact on veterans’ mental health and on treatment engagement and success.^11^ US veterans have unique mental health needs compared with the general US population, including relatively high rates of suicide, post-traumatic stress disorder (PTSD), depression and substance use, among other concerns.^12,13^ Service members are frequently dissuaded from speaking up across settings and in particular from disclosing mental health symptoms and engaging in mental healthcare.^13^ Although US veterans are beneficiaries of the only nationalised healthcare programme in the USA, the Department of Veterans Affairs (VA) has undergone considerable scrutiny, which has decreased some veterans’ willingness to seek services.^14^ Therefore, this vulnerable and specialised group face specific barriers to getting their mental health needs met during active duty and after.

CDM facilitates disclosure and empowerment, identifying a promising method to increase help-seeking and effective care for veterans. Although there is an increased appreciation of CDM and person-centred mental healthcare by military bodies, it has been mostly related to PTSD, most of the work was conducted within the USA and implementation remains challenging.^15–19^ Facing the growing need to improve veterans’ mental healthcare, this commentary illuminates clinical challenges for implementing CDM in military settings, focusing on veterans in the USA with mental illness.

## Challenge 1: when military culture clashes with CDM

Military practice and culture are rooted in hierarchy and obedience to authority. Veterans have been trained to believe in the importance of hierarchy and encouraged to obey and comply with perceived authority figures. Valuing hierarchy clashes with the CDM process, where clinicians and patients are equal contributors to the decision-making process. Our ongoing research suggests that when veterans are presented with the principles of CDM, they fear the consequences of ‘disrespecting’ or ‘disobeying’ clinician judgement if they disagree with treatment recommendations.^20^ Veterans often prefer to silence themselves or disengage entirely from mental health treatment rather than state a differing opinion.

## Challenge 2: when military culture reduces comfort with help-seeking

Many veterans held leadership positions in the military, meaning they expect themselves to be strong, competent and able to take care of their own problems.^21^ Such beliefs easily translate to expectations that one should handle any mental health issues independently, reducing willingness to engage in mental healthcare.^21^ It also could lead to difficulty being vulnerable, disclosing symptoms or other signs of perceived ‘weakness.’^21^ The deep comradery developed among service members who serve together can exacerbate this problem, as veterans may struggle to disclose issues that could reflect poorly on other service members (e.g. military sexual trauma, conflicts between service members).

## Challenge 3: when military communication style mismatches civilian therapeutic context

Veterans have been trained to use a direct and clear communication style in the military,^22^ which differs from civilian communication,^23^ leading to a communication mismatch that significantly interferes with CDM. Civilian clinicians who lack appropriate training in military culture may interpret the normative military communication style as aggressive or offensive, focusing clinical efforts on changing the veteran's communication style (as it is perceived as aggressive) rather than on the veteran's treatment needs and goals.^20^ Some veterans with severe mental illness (SMI) have described fear of being perceived as too aggressive by clinicians, including being disrespectful, malicious, dangerous or ‘crazy.’^20^ Such fear can decrease veterans’ willingness to assert preferences and reduce disclosures of important experiences and symptoms – key components of CDM.

## Challenge 4: when military bodies do not offer sufficient treatment options

Implementation of CDM requires a participatory decision-making process that considers all possible options based on the patient's needs, preferences and values.^4,24^ However, military healthcare often has only a few treatment options, and sometimes only one. While the VA's investment in evidence-based practices is a benefit in many ways, there is not an appropriate evidence-based practice for every person and clinical situation. Clinical trials represent about 20% of people with schizophrenia-spectrum disorders.^25^ People from minority racial/ethnic groups, those experiencing comorbid substance use, somatic disease and suicidality are (among others) less likely to be included, decreasing applicability of these treatments for many veterans with SMI.^25^ Therefore, owing to lack of treatment options, true CDM is limited within the VA setting, even when veterans and clinicians are fully committed to it.

## Overcoming these challenges

### Recommendations for clinicians

Civilian clinicians working with veterans should engage in training to increase their understanding of military culture and its impact on veterans’ interactions with mental healthcare. Engaging in mental healthcare and CDM means adapting processes to fit each individual's needs and values, including considering cultural identity. Significant literature supports the importance of military cultural competence^26^ among clinicians, including ability to increase treatment-seeking.^27^ It is crucial to understand a veteran's beliefs about their military service and how these beliefs shape their relationship to decision-making in mental healthcare. Attention to challenges of readjustment to civilian life have also been identified as particularly important when communicating with veterans.^28^ Clinicians should familiarise themselves with normative military communication. If needed, structured training should be completed to address the communication mismatch between veterans and civilian clinicians, so that clinicians adapt their clinical response appropriately and ensure that veterans’ treatment needs and goals remain the target of treatment rather than the clinician's perception of rudeness or impaired social skills.

Clinicians should encourage and empower veterans to participate in CDM, including psychoeducation about CDM, transparency about available treatment options, even if very few exist, jointly setting recovery goals and asking the veteran about preferred treatment options.^7^ The continual development of rapport, trust and transparency is fundamental to creating a therapeutic space supportive of CDM.^4^ A valid, CDM-aligned choice is for a veteran to decline to participate in a treatment decision.^4^ Clinicians are encouraged to become familiar with power-sharing in clinician–veteran dyads and consider how they can effectively empower veterans and create an egalitarian relationship. Positioning decision-making as shared risk-taking can help develop this understanding.^24^

CDM is a significant change from past interactions with providers, as veterans are often unaware that they have the option to collaborate in treatment decision-making. Veterans may need support to access CDM and effectively engage in it. We recommend empowerment-oriented, patient-centred strategies focused on increasing veterans’ knowledge, skills and confidence with engaging in CDM.^4^ For example, one CDM-aligned intervention adapted for the VA, collaborative decision skills training,^20^ includes fundamental CDM psychoeducation, communication and problem-solving skills, and brief coping skills to increase comfort and confidence. Interventions that address how CDM and self-advocacy broadly benefit veterans may also be helpful.^29^

### Policy and implementation recommendations

Military bodies must identify policies and changes that increase veterans’ access to options and consider how civilian clinicians and veterans communicate and share power. Although the specific actions will vary by country, the fundamental principles of prioritising veterans’ power over their care is essential: for example, broadening access to a variety of care options; expanding accessibility, including in rural areas and through telehealth, in-home and in-community options; investing in culturally tailored and trauma-informed approaches; and engaging veterans at all levels of decision-making, including policy decisions. For example, the USA's VA MISSION Act of 2018 intends to increase access to care for veterans under certain conditions (e.g. if a needed service is unavailable at the VA). Expanding community care can increase veterans’ power in decision-making by increasing choice over whether to receive care within the VA or the community. Such change can increase access for veterans who prefer community care and for veterans seeking culturally tailored care that the VA currently does not provide.

Military bodies should also invest in broad training and implementation of CDM. More intensive training is needed to customise to specific clinic and population needs and provide education about military culture and communication. More extensive training would also increase clinicians’ ability to assess and reflect on ways to step back from power and build trust.

Finally, investment in scholarships and infrastructure to diversify the clinician workforce is needed. The existing structure for licence-eligible clinical training can be difficult for veterans to access, especially veterans with lived experience of mental health concerns. A diverse clinical workforce facilitates the ability of veterans to work with clinicians with important shared experiences, improving effective tailoring for each veteran's needs and preferences and potentially increasing trust and collaboration for CDM. It also may increase awareness of military culture, and other lived experiences, in the clinician workforce, facilitating culturally informed care regardless of an individual clinician's background.

## Data Availability

Data availability is not applicable to this article as no new data were created or analysed in this work.
